# Supplementary material for: Pharmacoinformatic Investigation of Silymarin as a Potential Inhibitor against Nemopilema nomurai Jellyfish Metalloproteinase Toxin-like Protein
Source: Int J Mol Sci. 2023 May 18;24(10):8972. doi: 10.3390/ijms24108972 (PMC10219531; doi:10.3390/ijms24108972)
Supplement: Supplementary file 1 [file ijms-24-08972-s001.zip › supplementary file.pdf]

Supplementary Materials

Pharmacoinformatic Investigation of Silymarin as a Potential Inhibitor against *Nemopilema nomurai* Jellyfish Metalloproteinase Toxin-like Protein

Ravi Deva Asirvatham [1], Du Hyeon Hwang [1,2], Ramachandran Loganathan Mohan Prakash [1], Changkeun Kang [1,2] and Euikyung Kim [1,2,*]

[1] College of Veterinary Medicine, Gyeongsang National University, Jinju 52828, Republic of Korea; devabiochem@gnu.ac.kr (R.D.A.); pooh9922@hanmail.net (D.H.H.); mohanprakash111@gmail.com (R.L.M.P.); ckkang@gnu.ac.kr (C.K.)

[2] Institute of Animal Medicine, Gyeongsang National University, Jinju 52828, Republic of Korea

* Correspondence: ekim@gnu.ac.kr; Tel.: +82-55-772-2355; Fax: +82-55-772-2349

Contents:

- Sequence information of modeled NnV-MpI retrieved from *Nemopilema nomurai* tentacle transcriptome.
- Figure S1 (a, b, c, and d)
- Figure S2 (a, b, c, and d)
- Figure S3 (a, b, c, and d) and (a′, b′, c′, and d′)
- Figure S4 (a, b, c, and d)
- Figure S5 a and b (i, ii, iii and iv) and (i′, ii′, iii′ and iv′)
- Figure Legends

Sequence information of modeled NnV-Mpl retrieved from *Nemopilema nomurai* tentacle transcriptome.

- NODE_22415_length_2148_cov_32.291084_g6860_i3.p1 (Nnv-Mpl type- 1)

MNHLRCLQCLVALLFFGLSQLEAVSPEGELQGSREQSQLETEIGTKAGVKRRFRRQSSKYARLLFVNLSGREVYIMYMDYYRTLIRHKTLRRNEKRQLTTRIHTTWYAYDAVNREYMNINGKYNHLVRRS
QAGKETELMIKAKDGKTVDPPKLPSTDKYIEVMVIADSTVYKKRGNLTKKYVLTLMNAAKQLFYHKTFVVKLHLTVVRVMIIKNEQDDFKIVPYNQRRSMGNACNFAAAQNTLSELDDGHFDFAVLLTR
RPFGHTGYAPLFQMCNRRHACALVYDKGLAVSHVVAHEIGHSLGIEHDGLYNDCQRDGLLGSIMGKVLWSRFDRFYWSKCSQHQLKMTIGYFYCLDDEPVANITDYMEKLPGVYMNMDEQCQAAY
GSGYGRCNGYYECKHTYCTVAGRDINRDCFTRSRPPLEGTSCDKGKGWCIRGKCYPTGRLRPAPVHGHWSKWSSWGSCSVECGTGVITRRRSCDSPRPKHGGKTCVGEDSELKVCKRKKCKVAKSYID
ERNHHCQSMYGDSWVWFSRKRSEELVSSLSGLNCSFESNCSWTQFDRDD

- NODE_8677_length_4065_cov_15.681613_g1415_i5.p1 (Nnv-Mpl type- 2)

MDGVILTNEDQYLIKPLGRNNHQNAHVIVRRILPKATYQYHKEQLRVSAQGDHRARQKRSSRETSTEKTIETLLVVDQKMTQHYGLDGARQYALTVANNAHDILRDASIGPHPVNLVIVKILVLVAPQE
NLNINHHAHNTLASFGEWAWQLSKSLKAKGEDFDYALLLTRYDICKDKEQPCDYNGASYIGGMCNFPTSTAVIQDFGLITGFSIAHHIGHSLGMEHDRLGTSCYAENRVMKPNAGYGEGAFLWSSCSV
QKFQEFLRTDRSRCLDDVPPKTKQDNPAPLRNSGNVYDVVSQCELFGGKGSKGCYYGDKDEVCVELQCILQNATSCARTGKPPADGTECGAGKWCKHGRCIEQHTTDFSIVDGGWSSWGEFKSCSR
PCGGGVAYRERICRSPAPLRGGKYCEGDSKGYTICNNKPCPDSIMNFRKQQCKKQSGSFWGDILYNWTYLKTSGEKGTSMESSCMLECVAEKDSKVTKNFGRSVDGTDCFLYDMNLLGKCIAGKCVSV
GCDMKLGSREKVDRCGVCEGDGGSCRDEFEPEFHHAPTVFLNVTDRKSNVTEGLSGSLNITVKVNSTIQPNITASGAINGTNGNNTKVETKGINAAKFDNTTGTDHLLNTLRKLKVLRNLMKAVLQKRC
NQNKKCLARYKYFDLRRKKSEISRGRTFEWVTVSTGCSVTCGIGTKSLLSECRRVDDGSPVGRKFCSLLPEPVSRVRACKMPSCIARWAVSDWGSCTKTCGAGIQAREVFCIKTINEDFFVPDSLCNSARK
PVARRICNKRTCPPIWVTEPFGKCSTSCGRGVQLREVFCKSYVQGTGLAIVPDSRCDAAERPQRDLLCNVHNPCPGQGQSRI

- NODE_41303_length_745_cov_1.141369_g19525_i0.p1 (Nnv-Mpl type- 3)

MLNDLGPRALEIYRAQPRNWLIPRETRYVELYVVADSQEFQKLGSREAVRQRVLEVVNHVDKLYQELSFRVVLVGLEIWNKDKFYISRYANVTLENLLSWREQNLQGQHPHDNVQLITGVDFIGSTVGL
AKVSALCSRHSGAVNQDHSKNSIGVASTMAHELGHNLGMSHDEDIPGCYCPEPREGGGCIMTESIGSKFPRIFSRCSKIDLESFVTKPQTGCLTNVPDVNRFVGGPVCGNLFVEHGEQCD

- NODE_8373_length_4132_cov_652.221237_g1984_i1.p1 (Nnv-Mpl type- 4)

MPKTSCYLFVQADWLFTDKFAKSRVRALYLMTQYVQSVNSIYKATSFTGFQHNVTLKISRMKAQTQAECNADAGCKFKDENIGVAKFLELNSETDHDQHCLAYVFTYRDFDDGVLGLAWIGEASGAS
GGVCERWKPYAGGKYKSLNTGVVTTLNYQQDVPTKVTEITLAHEIGHNFGSQHDPVSNSPECSPGSAGGGNFIMYPRATSGSDSNNYKFSKCSRDYIGPVLNAKADICFKESDGPICGNKIVEKDEDCD
CGYEDDCKSERCCNKYVPKSNNNCKYNTTNAKSPTKCSPSEGPCCDGKTCSYGGAIGKNCSSETECAGKAACDGSSAKCPSPPPKPDVNTTCQGGAKVCLNGTCSGSICLKTNQTECMCTAKEAYCDI
CCMDGSDKATCKSKTAVKSGKTVSLKLAPGSPCDNYLGYCDIFDKCRKVDAEGPL

Figure S1.

a

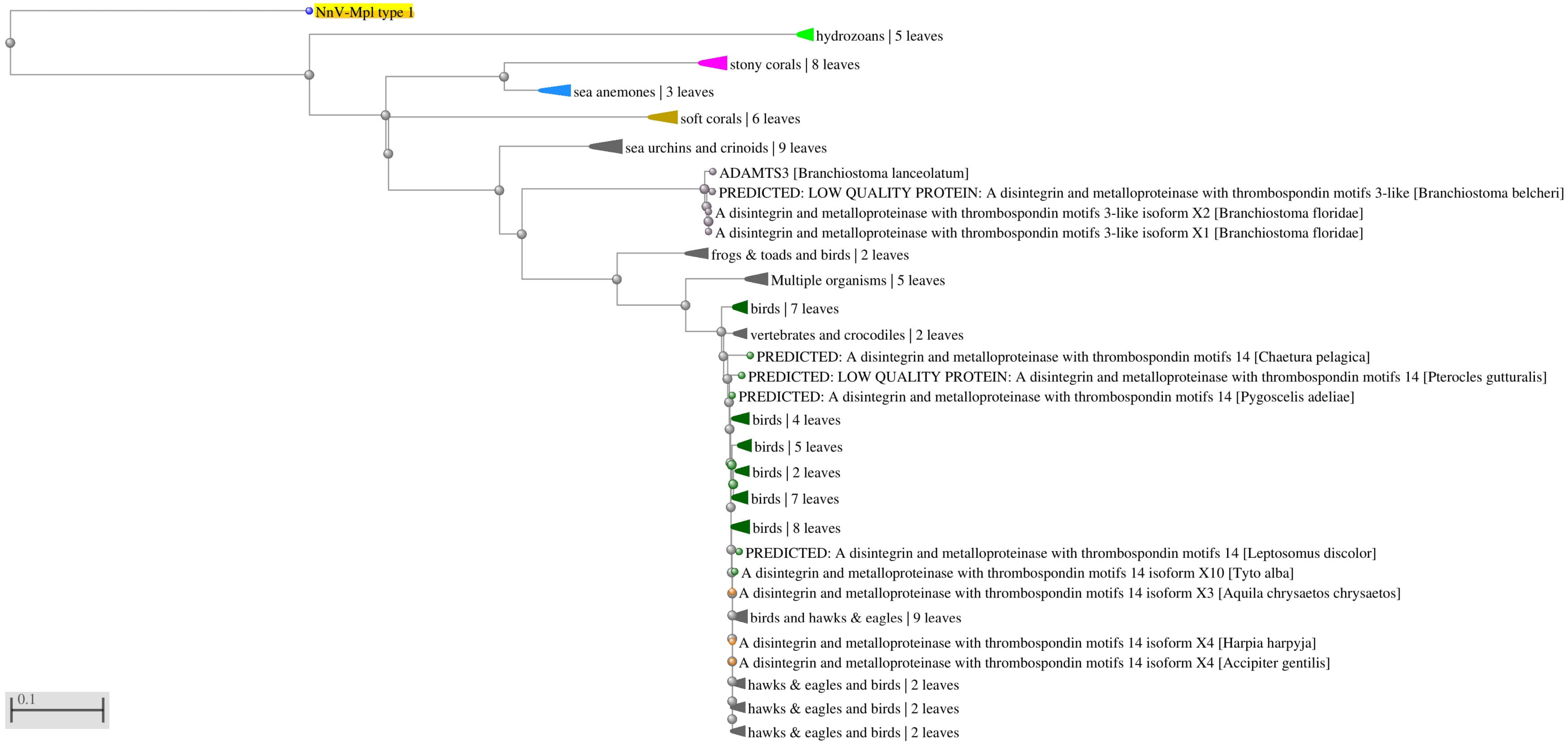

NnV-Mpl type 1
hydrozoans | 5 leaves
stony corals | 8 leaves
sea anemones | 3 leaves
soft corals | 6 leaves
sea urchins and crinoids | 9 leaves
ADAMTS3 [Branchiostoma lanceolatum]
PREDICTED: LOW QUALITY PROTEIN: A disintegrin and metalloproteinase with thrombospondin motifs 3-like [Branchiostoma belcheri]
A disintegrin and metalloproteinase with thrombospondin motifs 3-like isoform X2 [Branchiostoma floridae]
A disintegrin and metalloproteinase with thrombospondin motifs 3-like isoform X1 [Branchiostoma floridae]
frogs & toads and birds | 2 leaves
Multiple organisms | 5 leaves
birds | 7 leaves
vertebrates and crocodiles | 2 leaves
PREDICTED: A disintegrin and metalloproteinase with thrombospondin motifs 14 [Chaetura pelagica]
PREDICTED: LOW QUALITY PROTEIN: A disintegrin and metalloproteinase with thrombospondin motifs 14 [Pterocles gutturalis]
PREDICTED: A disintegrin and metalloproteinase with thrombospondin motifs 14 [Pygoscelis adeliae]
birds | 4 leaves
birds | 5 leaves
birds | 2 leaves
birds | 7 leaves
birds | 8 leaves
PREDICTED: A disintegrin and metalloproteinase with thrombospondin motifs 14 [Leptosomus discolor]
A disintegrin and metalloproteinase with thrombospondin motifs 14 isoform X10 [Tyto alba]
A disintegrin and metalloproteinase with thrombospondin motifs 14 isoform X3 [Aquila chrysaetos chrysaetos]
birds and hawks & eagles | 9 leaves
A disintegrin and metalloproteinase with thrombospondin motifs 14 isoform X4 [Harpia harpyja]
A disintegrin and metalloproteinase with thrombospondin motifs 14 isoform X4 [Accipiter gentilis]
hawks & eagles and birds | 2 leaves
hawks & eagles and birds | 2 leaves
hawks & eagles and birds | 2 leaves
0.1

Figure S1 (cont.)

b

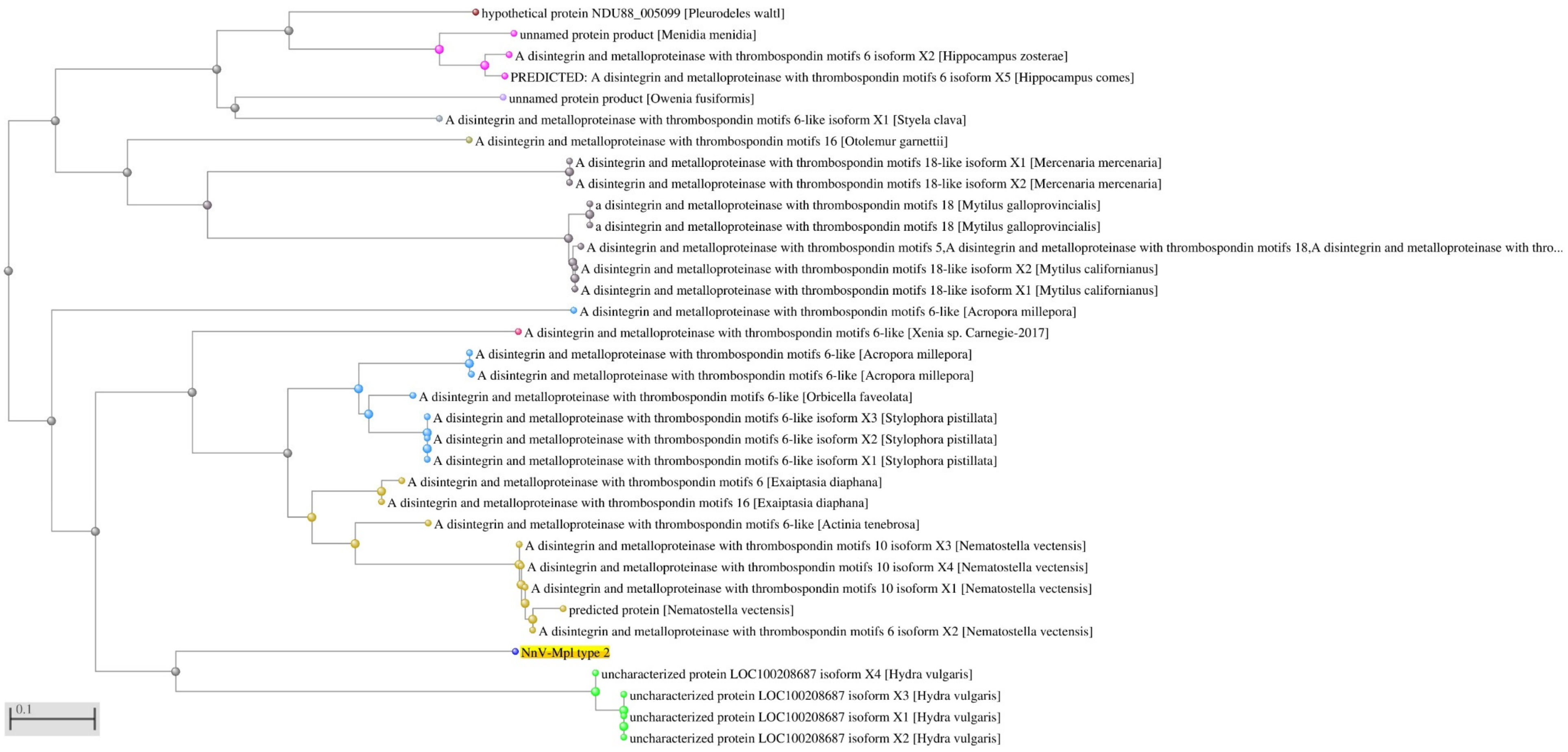

Figure S1 (cont.)

C

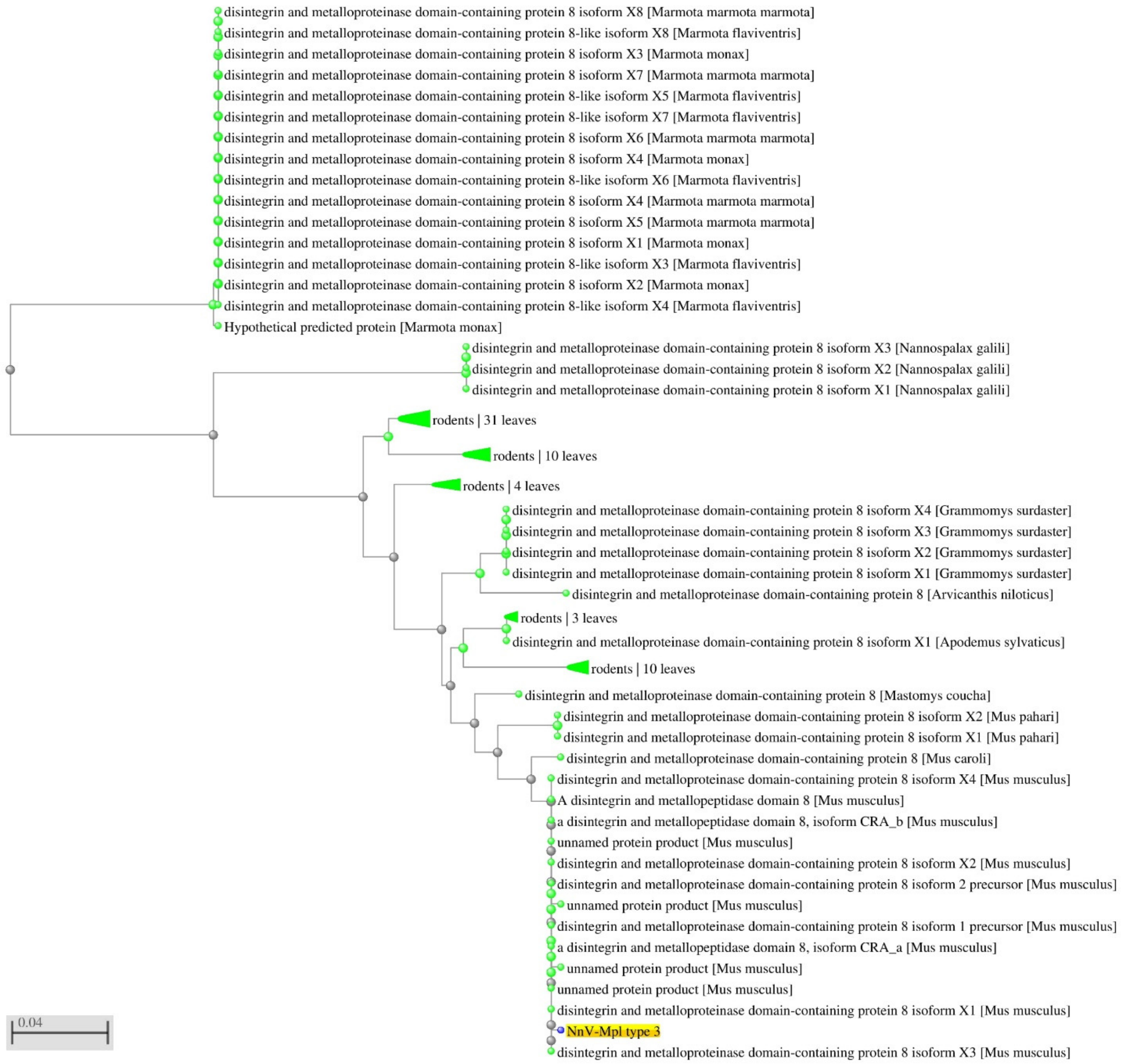

d

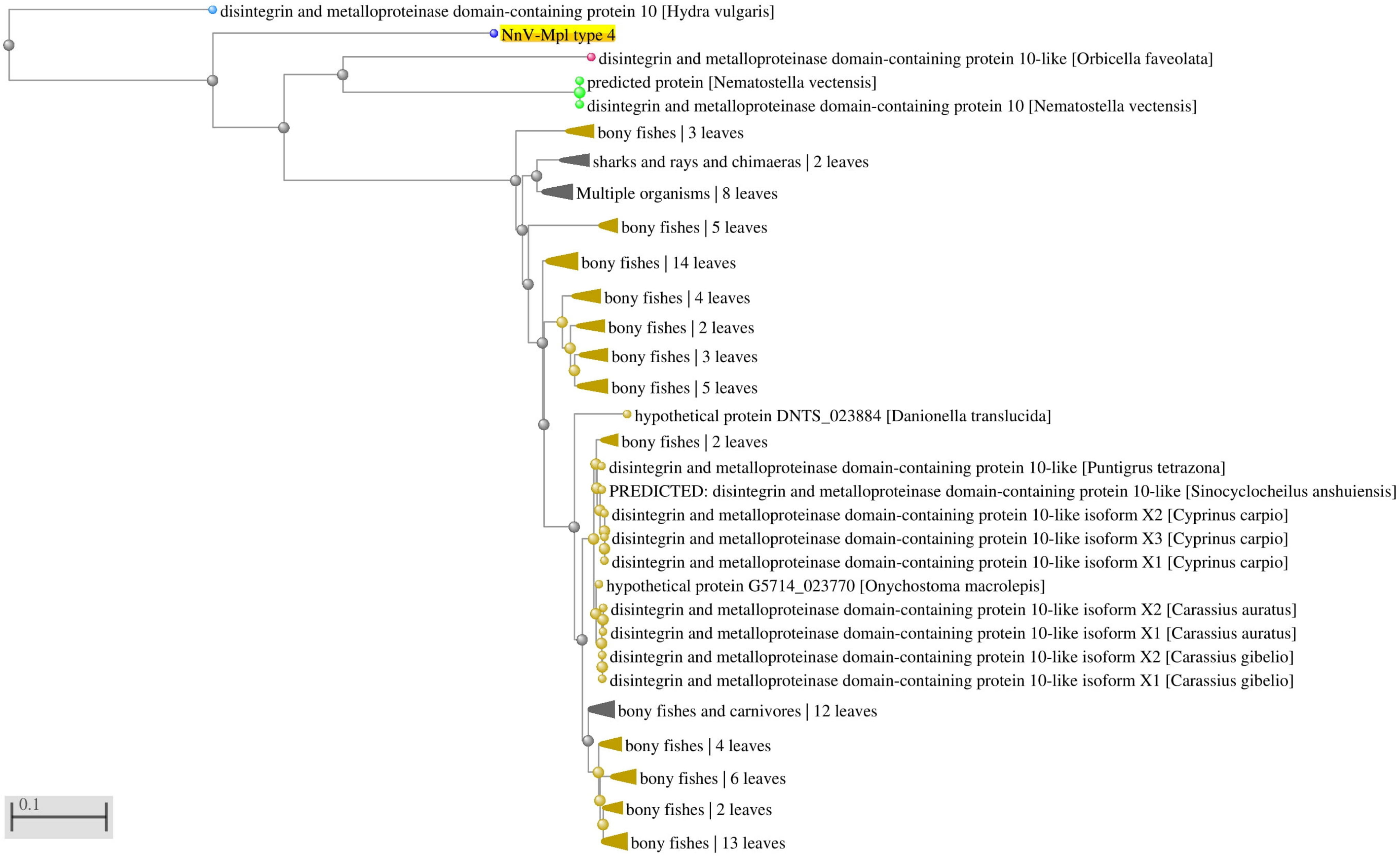
disintegrin and metalloproteinase domain-containing protein 10 [Hydra vulgaris]
NnV-Mpl type 4
disintegrin and metalloproteinase domain-containing protein 10-like [Orbicella faveolata]
predicted protein [Nematostella vectensis]
disintegrin and metalloproteinase domain-containing protein 10 [Nematostella vectensis]
bony fishes | 3 leaves
sharks and rays and chimaeras | 2 leaves
Multiple organisms | 8 leaves
bony fishes | 5 leaves
bony fishes | 14 leaves
bony fishes | 4 leaves
bony fishes | 2 leaves
bony fishes | 3 leaves
bony fishes | 5 leaves
hypothetical protein DNTS_023884 [Danionella translucida]
bony fishes | 2 leaves
disintegrin and metalloproteinase domain-containing protein 10-like [Puntigrus tetrazona]
PREDICTED: disintegrin and metalloproteinase domain-containing protein 10-like [Sinocyclocheilus anshuiensis]
disintegrin and metalloproteinase domain-containing protein 10-like isoform X2 [Cyprinus carpio]
disintegrin and metalloproteinase domain-containing protein 10-like isoform X3 [Cyprinus carpio]
disintegrin and metalloproteinase domain-containing protein 10-like isoform X1 [Cyprinus carpio]
hypothetical protein G5714_023770 [Onychostoma macrolepis]
disintegrin and metalloproteinase domain-containing protein 10-like isoform X2 [Carassius auratus]
disintegrin and metalloproteinase domain-containing protein 10-like isoform X1 [Carassius auratus]
disintegrin and metalloproteinase domain-containing protein 10-like isoform X2 [Carassius gibelio]
disintegrin and metalloproteinase domain-containing protein 10-like isoform X1 [Carassius gibelio]
bony fishes and carnivores | 12 leaves
bony fishes | 4 leaves
bony fishes | 6 leaves
bony fishes | 2 leaves
bony fishes | 13 leaves
0.1

Figure S2.

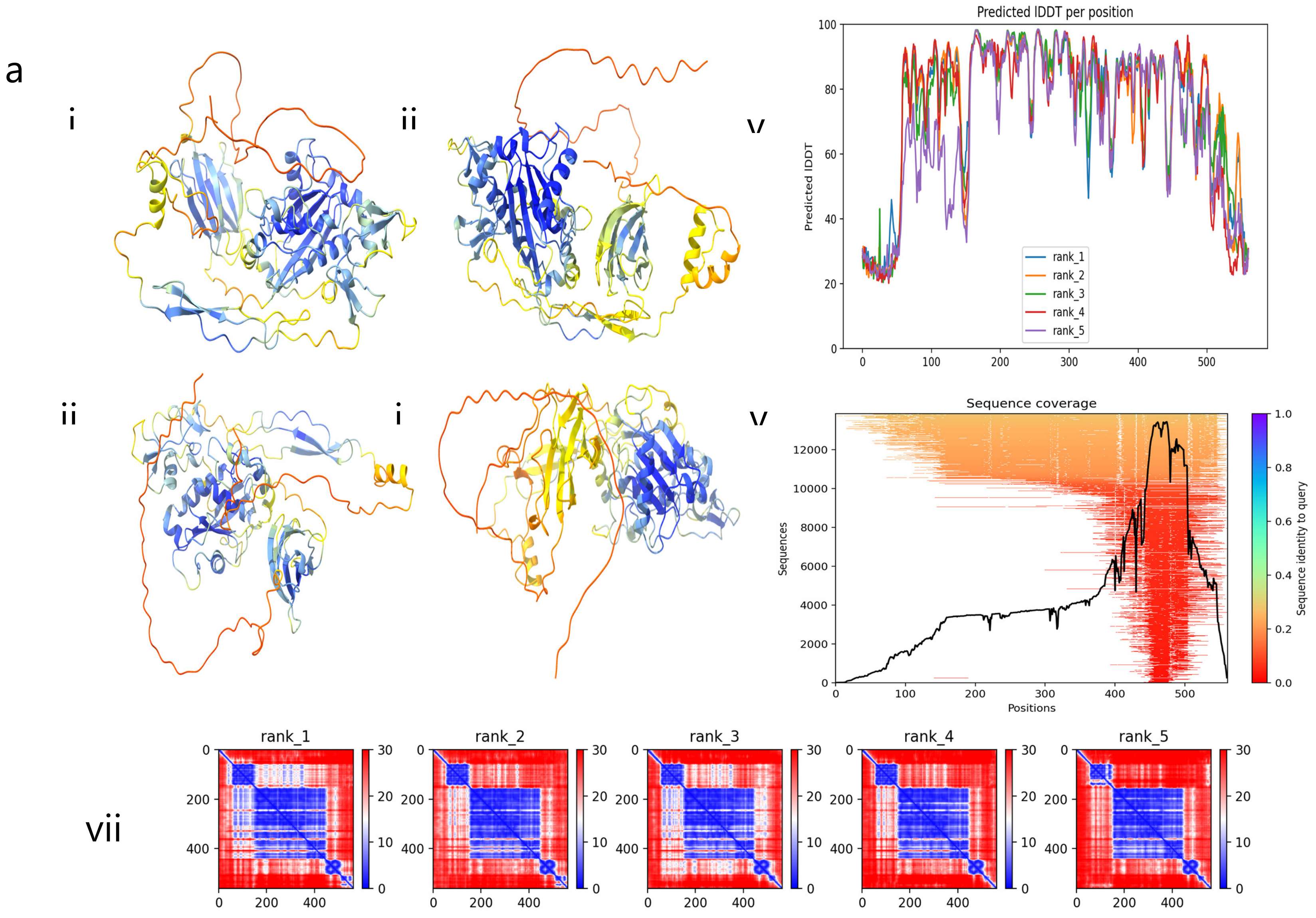

Figure S2 (cont.)

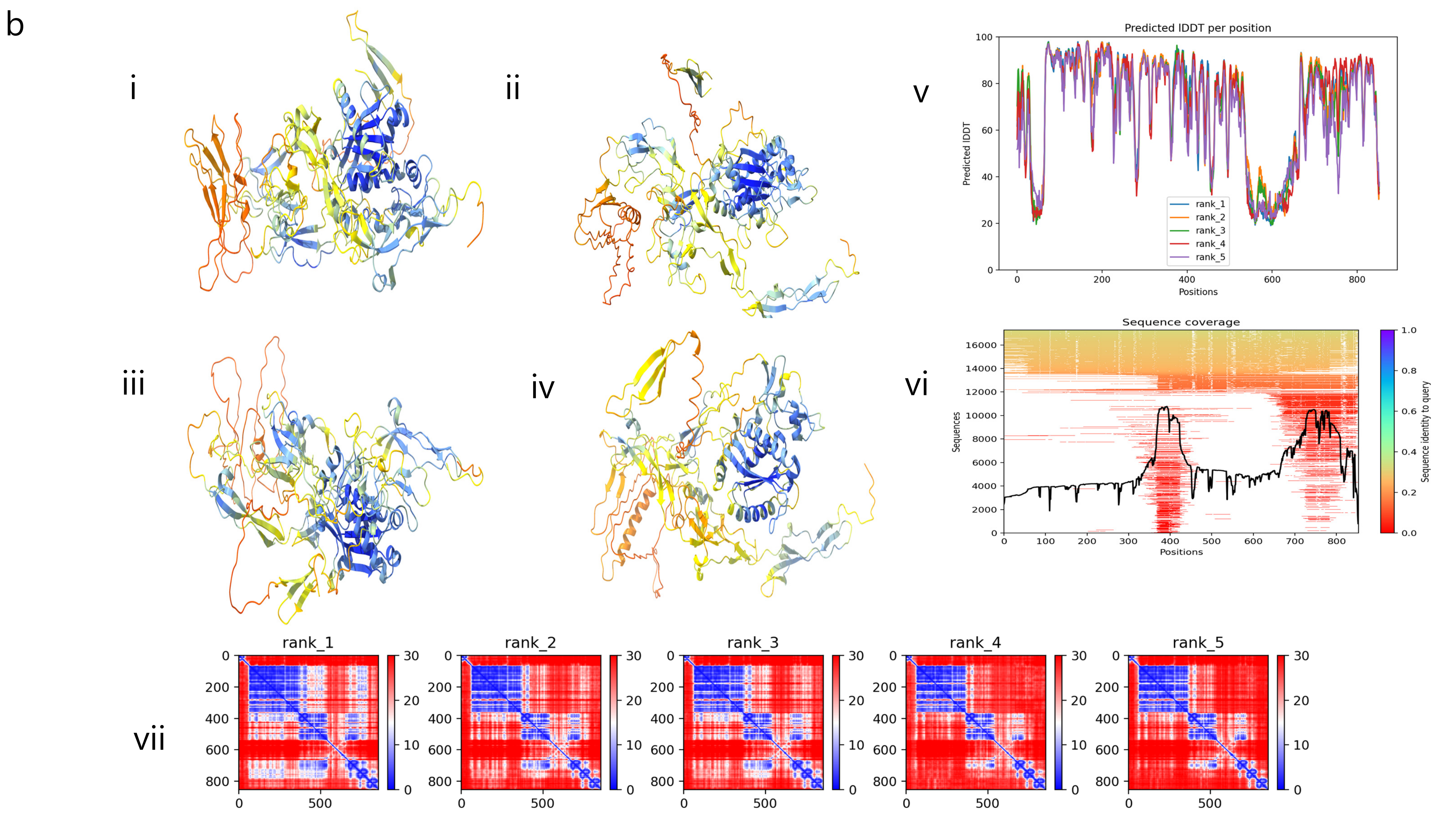

Figure S2 (cont.)

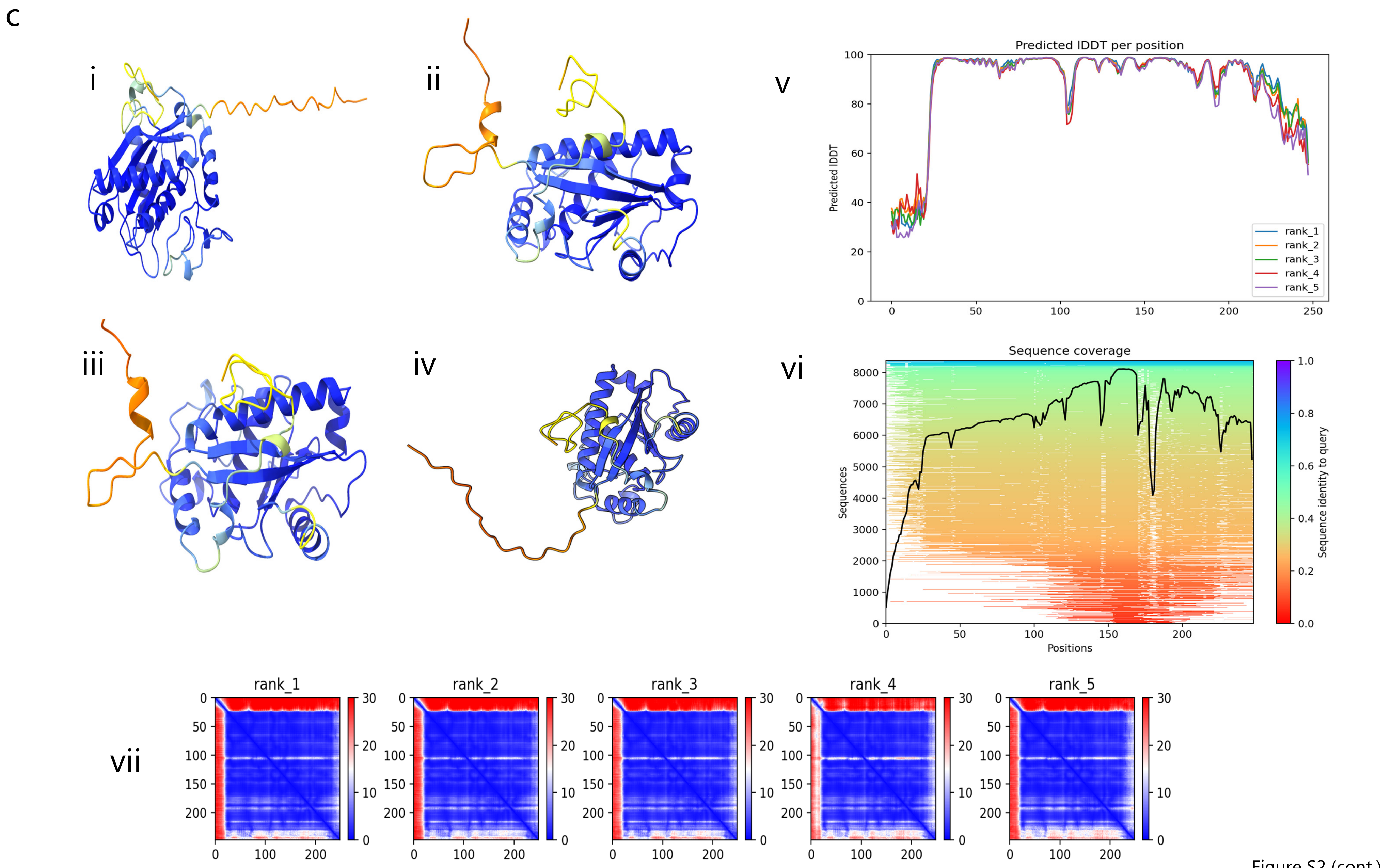

Figure S2 (cont.)

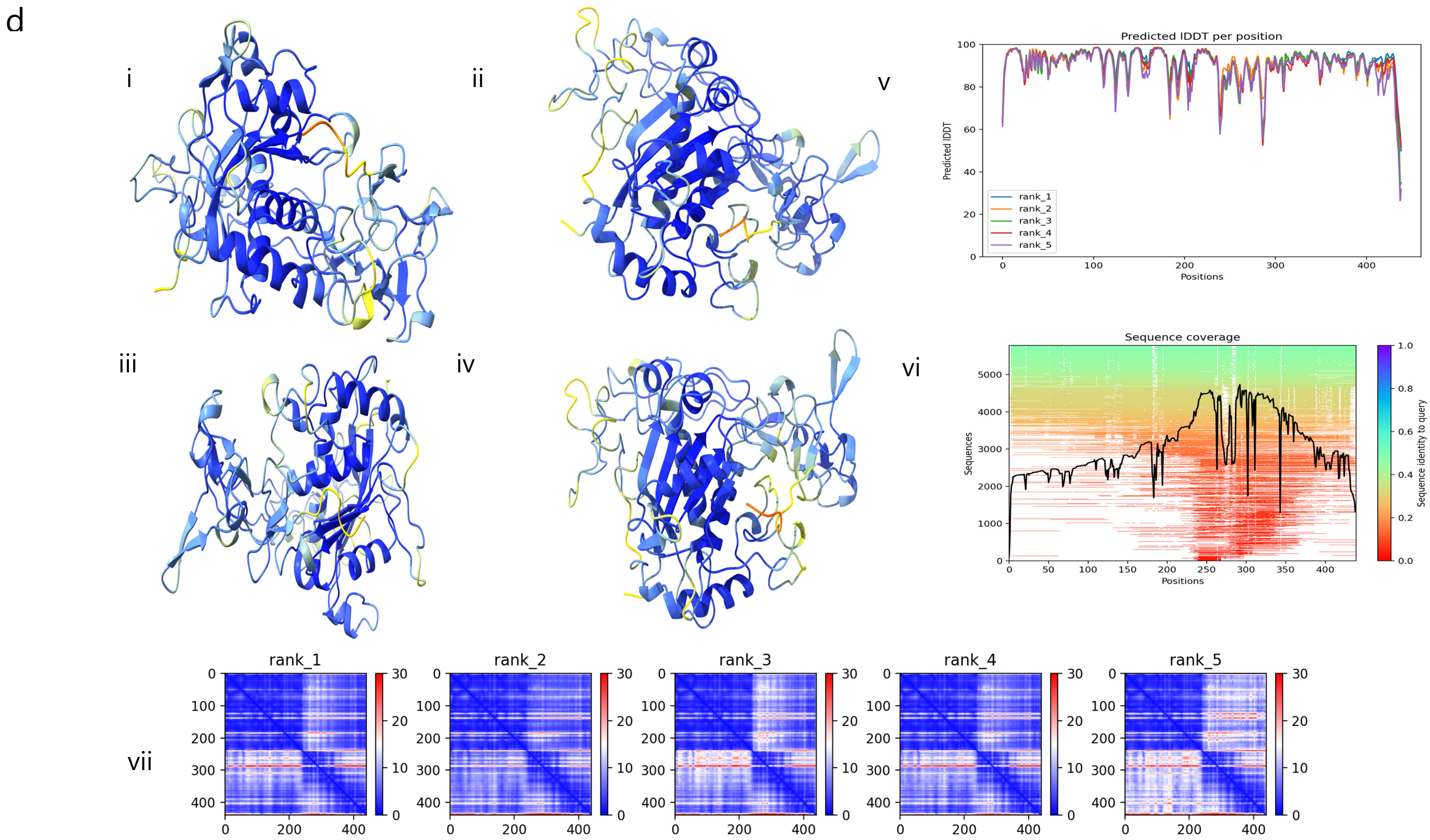
d
i
ii
iii
iv
v
vi
vii
Predicted lDDT per position
Predicted lDDT
Positions
rank_1
rank_2
rank_3
rank_4
rank_5
Sequence coverage
Sequences
Sequence identity to query
rank_1
rank_2
rank_3
rank_4
rank_5

Modeled protein	Score	Model 1	Model 2	Model 3	Model 4	Model 5
NnV-Mlp type 1	PLDDT	70.4	72.1	72.4	69.8	71.8
	ptm	0.463	0.439	0.448	0.471	0.455
NnV-Mlp type 2	PLDDT	70.4	67.2	71.3	69	62
	ptm	0.586	0.564	0.594	0.573	0.548
NnV-Mlp type 3	PLDDT	75	68.6	75.4	73.5	73
	ptm	0.621	0.585	0.654	0.642	0.604
NnV-Mlp type 4	PLDDT	89.2	90.3	91.7	90.9	90.9
	ptm	0.813	0.869	0.877	0.847	0.888

Figure S3.

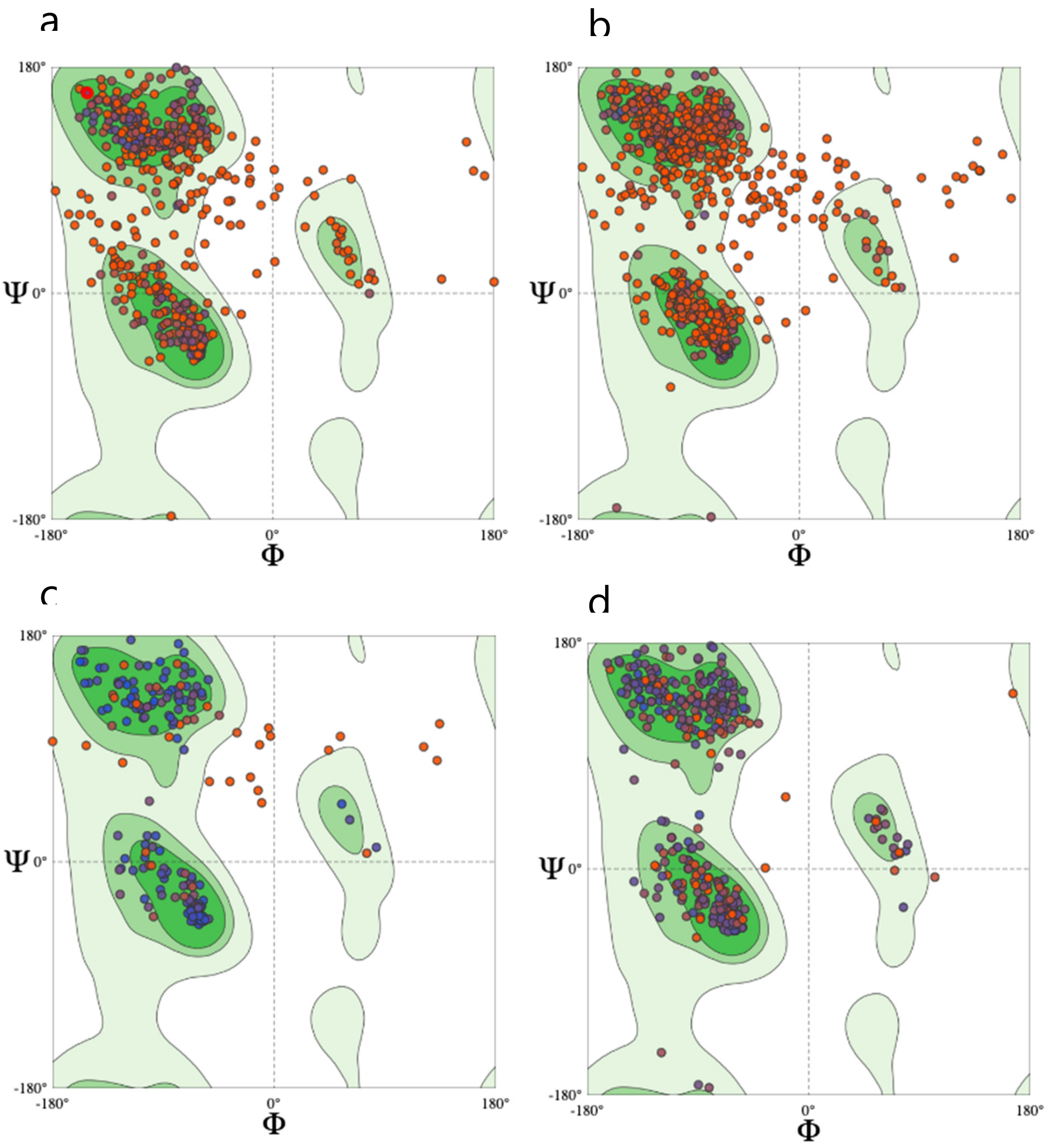

Figure S3 (cont.)

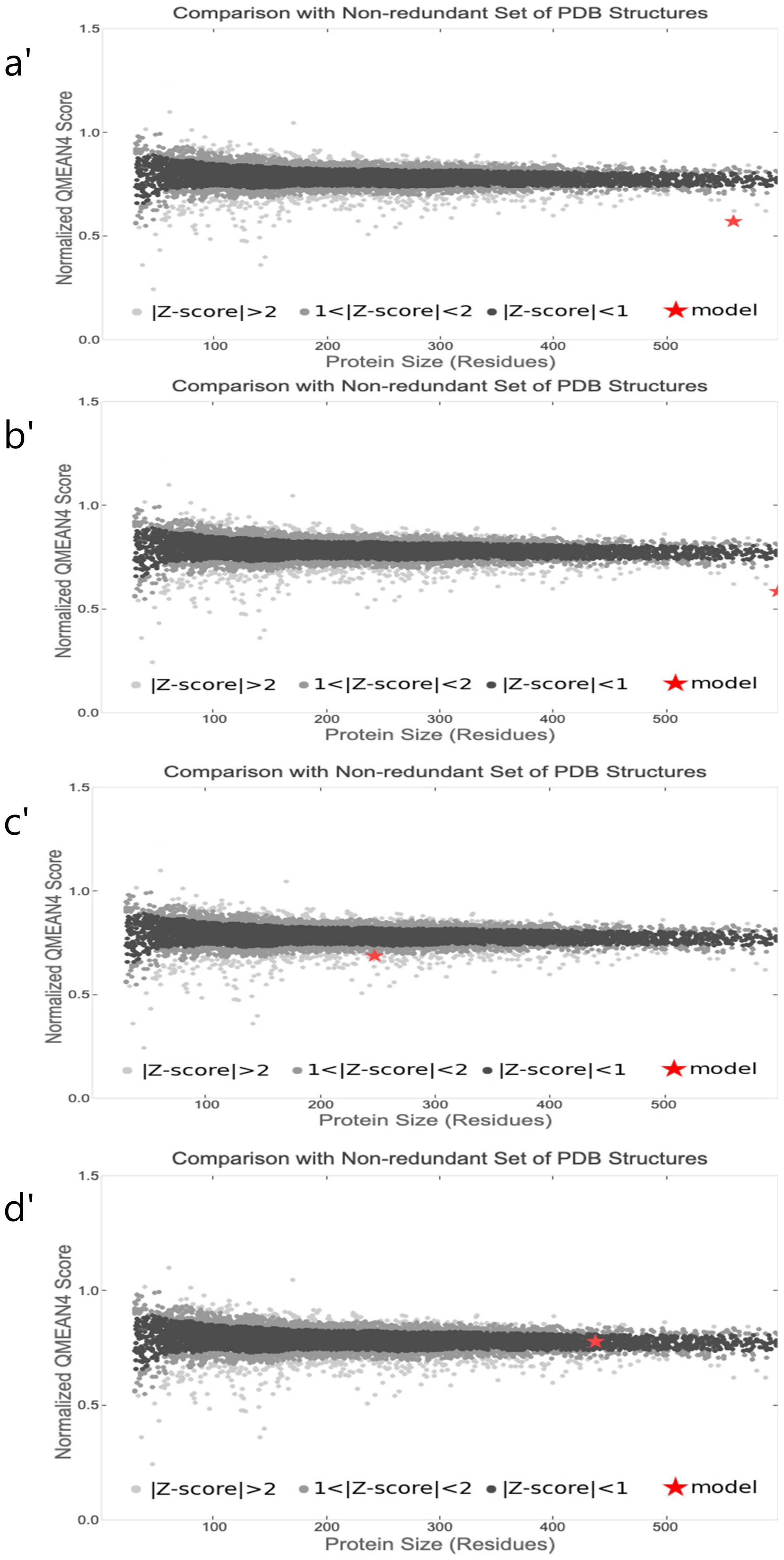
a'
b'
c'
d'
Comparison with Non-redundant Set of PDB Structures
Normalized QMEAN4 Score
1.5
1.0
0.5
0.0
100
200
300
400
500
Protein Size (Residues)
|Z-score|>2
1<|Z-score|<2
|Z-score|<1
model

Metalloproteinase	Ramachandran Favoured	MolProbity Score	Rotamer Outliers	C-Beta Deviations	QMEANDisCo Global
NnV-Mlp type 1	83.72	3.21	3.46%	0	0.46 ± 0.05
NnV-Mlp type 2	83.08	2.96	2.74%	0	0.47 ± 0.05
NnV-Mlp type 3	90.65%	2.32	0.46%	0	0.73 ± 0.05
NnV-Mlp type 4	95.65%	2.09	0.00%	0	0.67 ± 0.05

Figure S4.

a

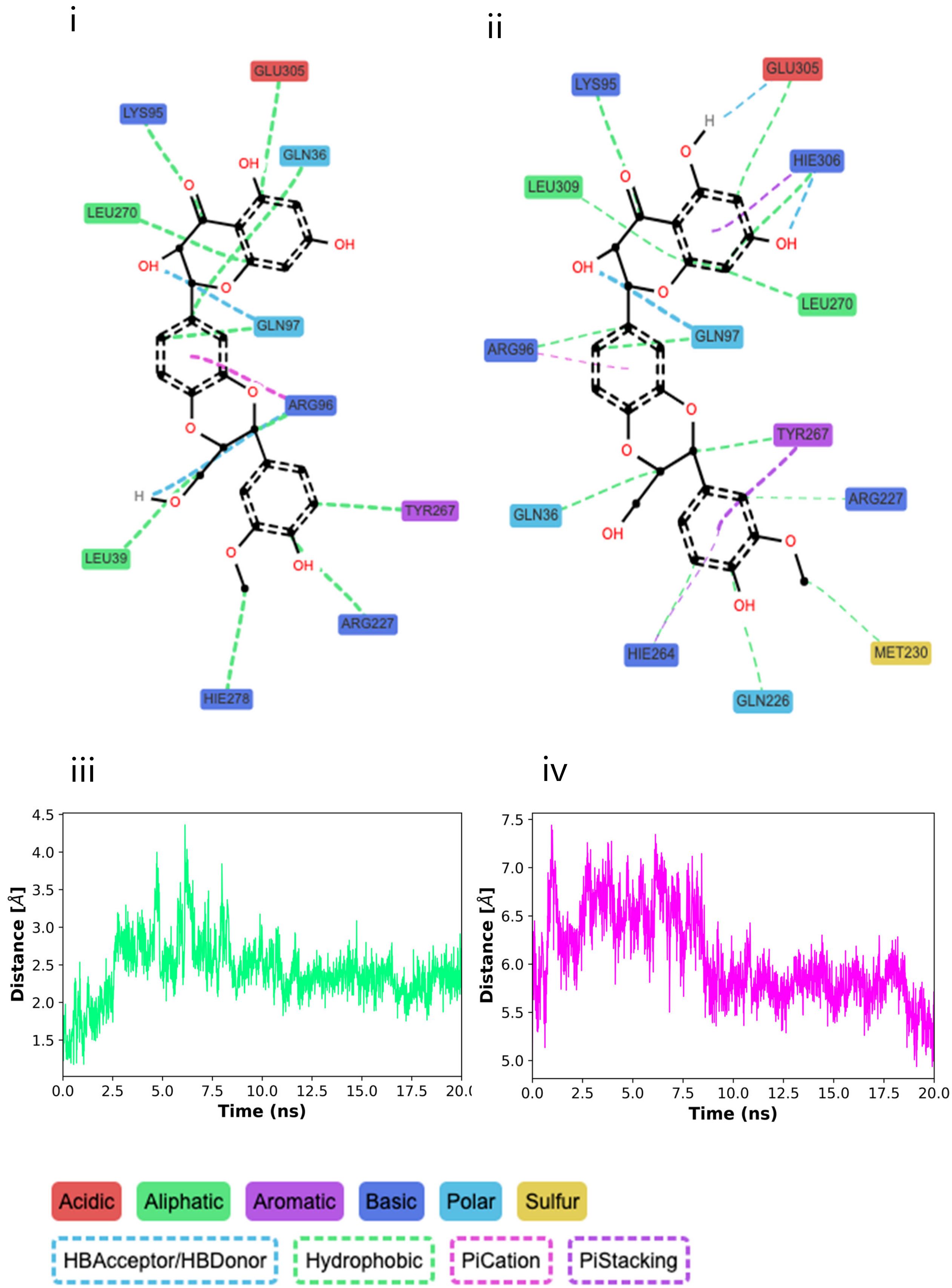

Figure S4 (cont.)

b

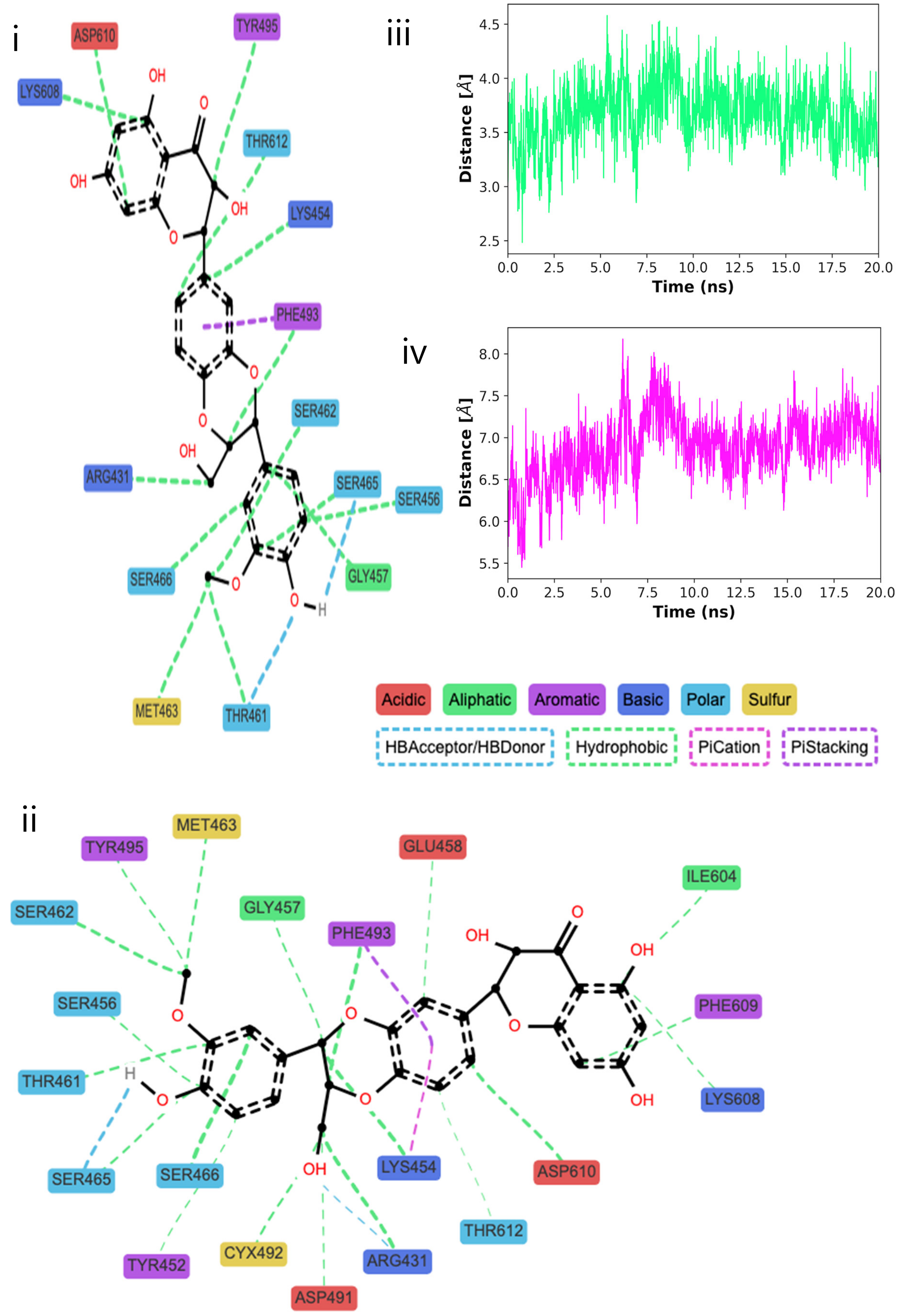

Figure S4 (cont.)

c

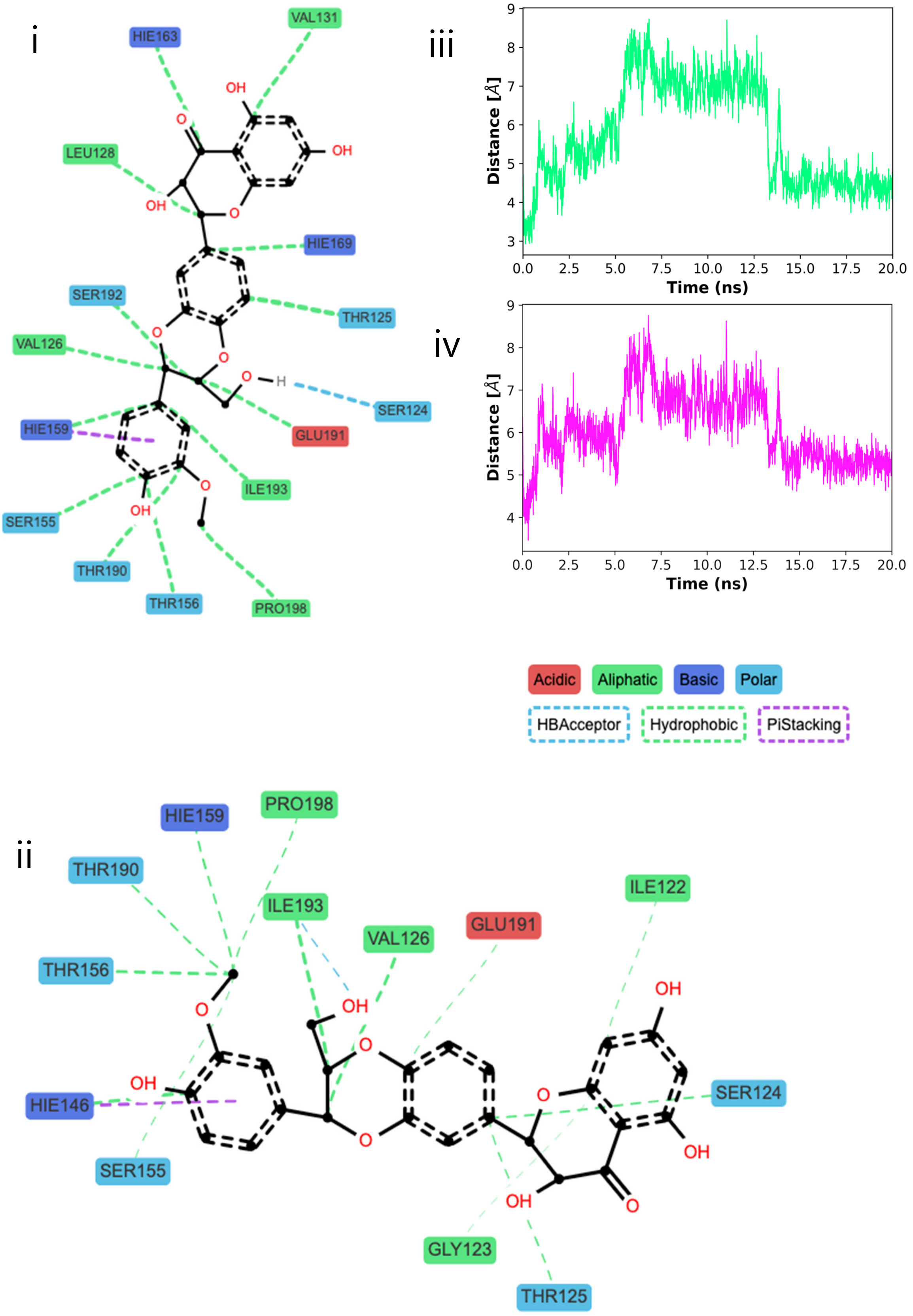

Figure S4 (cont.)

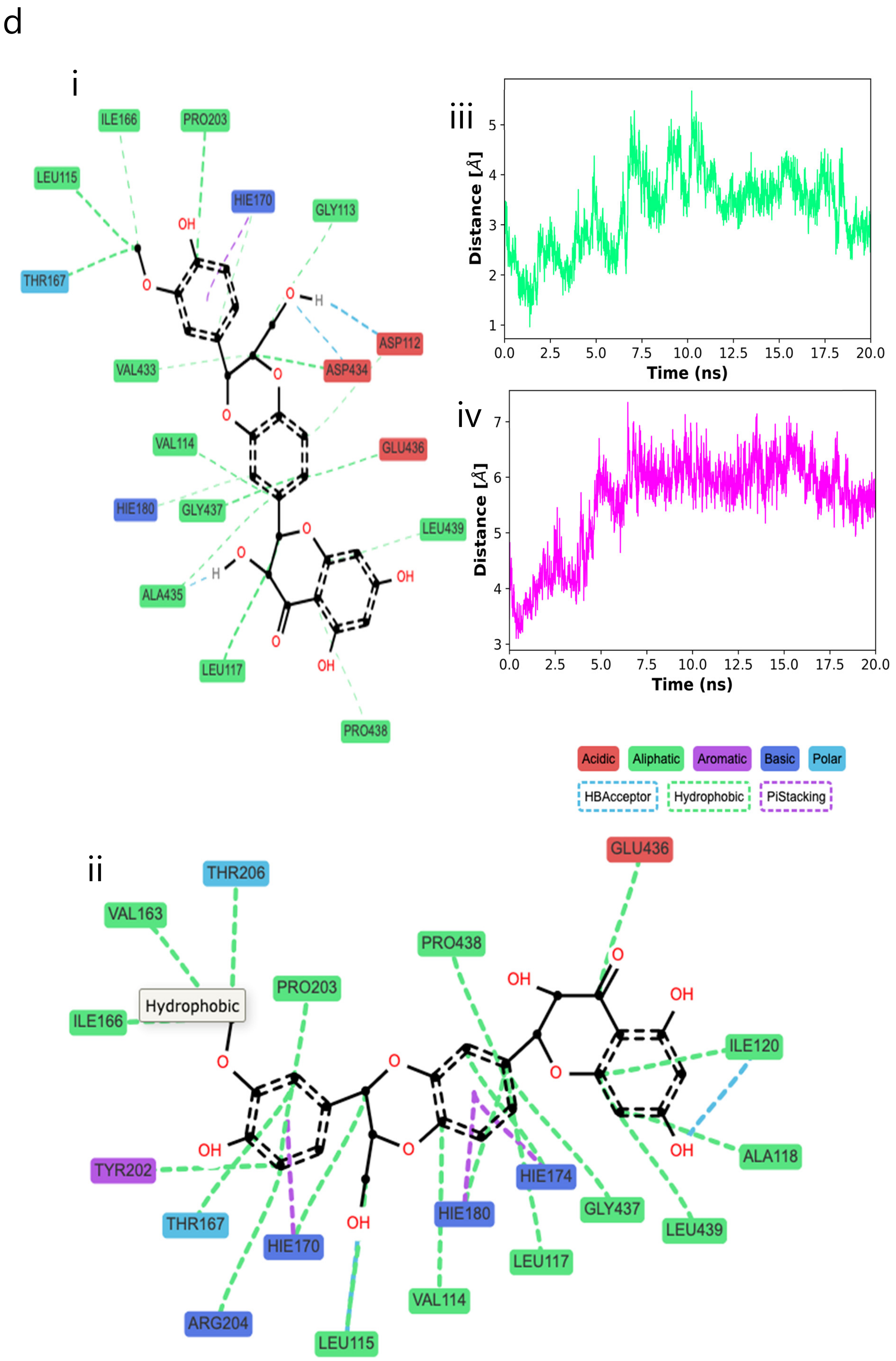

d
i
ILE166
PRO203
LEU115
HIE170
GLY113
THR167
ASP112
VAL433
ASP434
VAL114
GLU436
HIE180
GLY437
LEU439
ALA435
LEU117
PRO438
OH
iii
Distance [Å]
Time (ns)
0.0 2.5 5.0 7.5 10.0 12.5 15.0 17.5 20.0
1 2 3 4 5
iv
Distance [Å]
Time (ns)
0.0 2.5 5.0 7.5 10.0 12.5 15.0 17.5 20.0
3 4 5 6 7
Acidic
Aliphatic
Aromatic
Basic
Polar
HBAcceptor
Hydrophobic
PiStacking
ii
THR206
VAL163
GLU436
PRO438
PRO203
Hydrophobic
ILE166
ILE120
TYR202
ALA118
THR167
HIE170
HIE180
HIE174
GLY437
LEU439
LEU117
ARG204
VAL114
LEU115
OH

Figure S5.

a

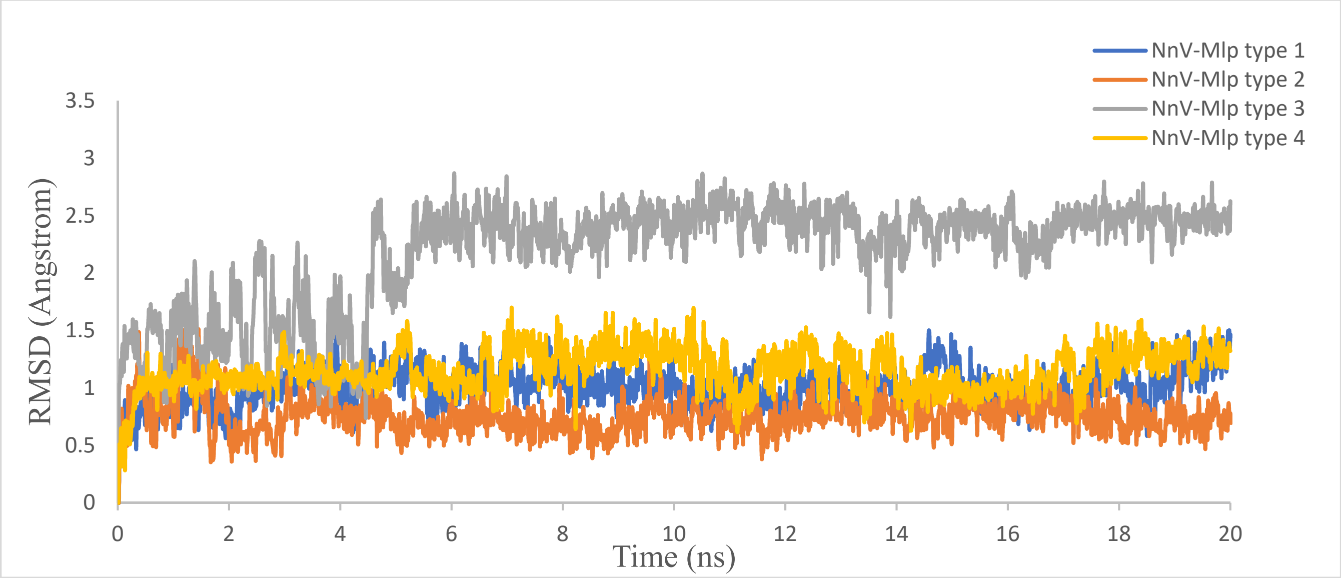

NnV-Mlp type 1
NnV-Mlp type 2
NnV-Mlp type 3
NnV-Mlp type 4
RMSD (Angstrom)
3.5
3
2.5
2
1.5
1
0.5
0
0
2
4
6
8
10
12
14
16
18
20
Time (ns)

b

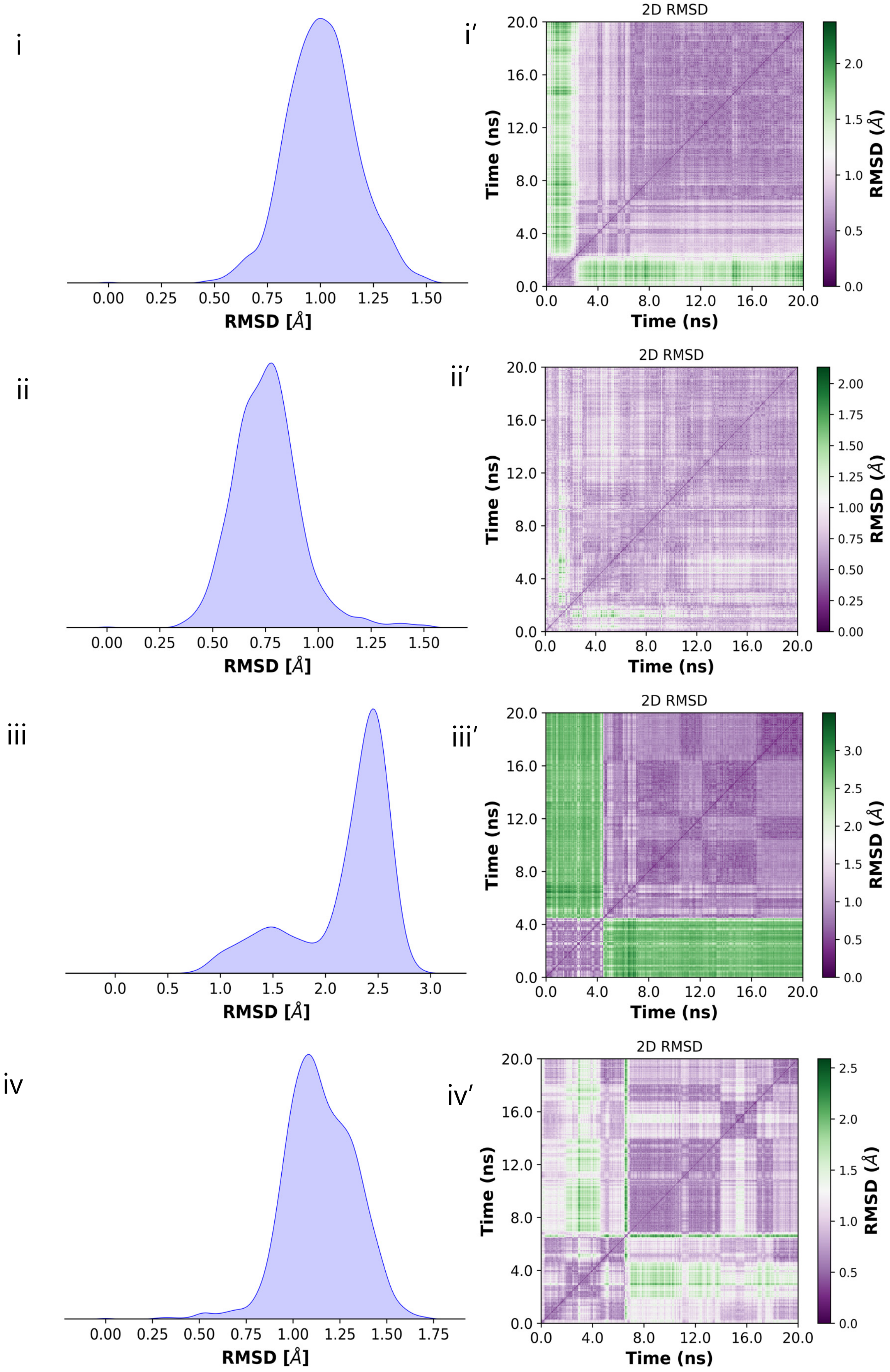

Figure Legends

Figure S1. The neighbor-joining method is a distance-based method for constructing evolutionary trees. constructing a Neighbor-Joining Tree for a quick look at our sequences and their relation to each other.
The below images show the relationship between NnV-Mpl and other similar metalloproteinase sequences.

Figure S2. Prediction of the protein complex structures by ColabFold and models were ranked based on AlphaFold pTM score (depicted in the table below the figure) (a) Type 1 NnV-Mlp; (b) Type 2 NnV-Mlp; (c) Type 3 NnV-Mlp; (d) Type 4 NnV-Mlp.

Figure S3. Ramachandran plot and QMEAN4 score (depicted in the table below the figure) for (a) Type 1 NnV-Mlp; (b) Type 2 NnV-Mlp; (c) Type 3 NnV-Mlp; (d) Type 4 NnV-Mlp and (a') Type 1 NnV-Mlp; (b') Type 2 NnV-Mlp; (c') Type 3 NnV-Mlp; (d') Type 4 NnV-Mlp respectively.

Figure S4. The distance of the ligand interacting with amino acid residues (a) Type 1 NnV-Mlp; (b) Type 2 NnV-Mlp; (c) Type 3 NnV-Mlp; (d) Type 4 NnV-Mlp. i- zero ns interaction with ligand; ii- 20 ns interaction with ligand; iii- calculation of ligand-amino acid polar residue distance over 20ns around 3-angstrom distance.; iv- calculation of ligand-amino acid residue distance over 20ns around 3-angstrom distance.

Figure S5

(a) Ligand RMSD of NnV-Mlp types were recorded for 2000 frames, (b) NnV- Mlp types (i), (ii), (iii), and (iv) correspond average distance of ligand RMSD for 20 ns and (i'), (ii'), (iii'), and (iv') denotes 2D RMSD of Ligand.
